# Retrospective study of combined splinting restorations in the aesthetic zone of periodontal patients

**DOI:** 10.1038/sj.bdj.2016.178

**Published:** 2016-03-11

**Authors:** X. Liu, Y. Zhang, Z. Zhou, S. Ma

**Affiliations:** 1 The First Affiliated Hospital of Jinan University, Guangzhou 510630, China

## Abstract

Aims This retrospective study evaluated the clinical performance of combining a fiber-reinforced, composite-resin (FRC) bonded splint; bridge; fiber post; and/or resin veneer for the restoration of periodontally-involved anterior teeth.

Methods Sixty-three patients with periodontally-involved anterior teeth each received one of the following splinting restorations: FRC-bonded splint-bridges (SB), a combination of an FRC-bonded splint and resin veneer (SV), or FRC-bonded splints (S). Six patients with healthy periodontal teeth received orthodontic retainers with FRC-bonded splints (OS) as a control. Modified USPHS criteria were used to evaluate the effects of rehabilitation, and the periodontal pocket depth (PPD) and clinical attachment level (CAL) of the natural teeth were measured.

Results All restorations resulted in good aesthetic outcomes. SV, S in combination with fiber posts and OS all resulted in 100% acceptable ratings for each category that was directly evaluated at each follow-up. The mean PPD was 3.5 ± 1.0 mm at baseline, decreased to 3.3 ± 1.0 mm after one year (p <0.05), and subsequently increased to 3.5±1.0 mm again after four years (p >0.05). These changes were similar to those in the mean CAL.

Conclusion A combination of a FRC-bonded splint, bridge, fiber post and/or resin veneer for minimally invasive prosthodontic treatment in the aesthetic zone is a good choice for periodontal patients.

## Abstract (hidden executive summary)

- Evaluates the clinical performance of of a fiber-reinforced, composite-resin bonded splint; a bridge; a fiber post; and/or a resin veneer for the restoration of periodontally-involved anterior teeth.
- Shows that a combination of approaches is a good choice for minimally invasive prosthodontic treatment in the aesthetic zone in periodontal patients.

## Introduction

The loss of a single anterior tooth may be a catastrophic event for a patient.^1^ In advanced periodontal disease such a loss is unfortunately often accompanied by adjacent tooth mobility due to severe bone loss. Furthermore, periodontal disease also chronically dislodges teeth due to the masticatory movement of the mobile teeth, which seriously affects anterior aesthetics, especially for the upper anterior teeth. Therefore, prosthodontic rehabilitation is often necessary to restore aesthetics and function in periodontally compromised dentition.^2^,^3^

Prosthodontic treatment for periodontally-involved anterior teeth usually includes dental implants, partial fixed dental prostheses (PFDPs), resin-bonded partial fixed dental prostheses (RBPFDPs), or partial removable dental prostheses (PRDPs).^2^,^4^ Implant restoration is the first choice for the treatment of suitable systemic and local conditions. However, as Donos *et al*. reported, the patient and site-risk profile should be assessed in combination with a cost–benefit analysis based on the patient's expectations for the implant treatment of periodontitis. This analysis should be followed by an extended observation period after the completion of periodontal therapy.^5^ Therefore, the majority of patients do not immediately choose implant prostheses. PFDPs are an alternative treatment that exhibit high mechanical strength and resistance to dislodgement but require full-tooth preparation.^4^,^6^ PRDPs may increase the torque of the abutting teeth, resulting in mobility. Therefore, many patients decline RBPRDPs in favour of a conservative alternative that requires less tooth reduction, less gingival disturbance, and reduced chair time.^4^,^7^,^8^ The fiber-reinforced, composite-resin (FRC) bonded splint-bridge is a type of RBPRDP that is combined FRC splinting adjacent to the mobile tooth. This approach can provide a simple, comfortable, affordable, non-invasive and aesthetic rehabilitation program^9^,^10^ for periodontally-involved anterior tooth loss. Extracted teeth or composite resins have been used for pontics.^11^,^12^ Usually, the FRC-bonded splint-bridge can be used as a long-term or temporary restoration for periodontal disease before or during implantation.^13^,^14^ However, few long-term clinical studies have examined outcomes for FRC-bonded splint-bridges in periodontally-involved anterior upper and lower teeth.^15^,^16^,^17^

The prosthodontic treatment of periodontally-involved anterior teeth with mobility and chronic dislodgement is often a dilemma when the extraction of dislodged teeth seriously affects anterior aesthetics. This challenge is especially common for severe labial or lingual dislodgement because the effect of rehabilitation is uncertain, irrespective of the extraction of dislodged teeth. Recently, adequate orthodontic and periodontal treatment has been sought to improve the periodontal condition and anterior aesthetics.^18^,^19^,^20^ However in such cases, periodontal breakdown causes pathologic migration of teeth, making the orthodontic treatment more complicated.^21^,^22^,^23^ At present, few clinical studies have examined the aesthetic restoration of periodontally-involved anterior teeth with mobility and chronic dislodgement. The development of silanised and resin-impregnated FRC materials has provided potential new approaches for stabilising hypermobile teeth or replacing teeth in a conservative manner.^24^,^25^ In this study, a combination of an FRC-bonded splint, resin veneer and/or fiber post was used to restore anterior teeth with mobility and chronic dislodgement without tooth extraction.

Therefore, this study systematically and retrospectively investigated anterior teeth restoration cases of periodontally treated patients restored with a combination of an FRC-bonded splint, bridge, fiber post and/or resin veneer to summarise the clinical operation and evaluated the clinical performance.

## Materials and methods

### Patient population

This study was approved by the Ethics Committee of the First Affiliated Hospital of Jinan University in Guangzhou, China. Between October 2009 and September 2014, 63 patients (36 females and 27 males, between 38 and 76 years old) with 63 units of anterior segment on one jaw received different splinting restorations at the Department of Prosthodontics of the Medical Center of Stomatology at the First Affiliated Hospital of Jinan University. Patients with periodontally-involved anterior teeth and anterior teeth mobility who had been diagnosed with chronic periodontitis according to the 1999 APP (American Academy of Periodontology) Classification^26^,^27^ were admitted to the study unless they met the following exclusion criteria: (1) they were younger than 18 years old; (2) they were unable to read and sign the informed consent document; (3) they presented with concurrent psychological disorders; (4) they suffered from general health-compromising conditions; (5) they were pregnant; (6) they were smokers; (7) they had history of restoration or whitening treatments for the teeth to be evaluated; (8) they could not or were unwilling to return for follow-up; (9) they had lost two or more teeth in the anterior segment of one jaw; or (10) one canine tooth was missing or both canines in the anterior segment on one jaw were mobile. According to the 1999 International Workshop Classification,^27^,^28^ 63 patients were diagnosed with severe chronic periodontitis. These patients had to undergo initial periodontal therapy, including oral health instructions, scaling, root planning, and local drug treatment. Scaling and root planning was implemented at intervals of four to six weeks until the patient's oral hygiene was stable, which was indicated by the absence of bleeding on probing.^29^,^30^ The patients were educated about oral hygiene protocols and recalled for periodical follow-up controls annually after splinting. All teeth with FRC bonding that presented with a mobility of MII or more [mobility – classified according to Miller's classification]^31^ after initial periodontal therapy or severe facial-lingual dislodgement received root canal treatments and fiber-post restorations.

In addition, six patients (three females and three males, between 18 and 28 years old) who were free of periodontal disease received orthodontic retainers with FRC-bonded splints to compare the effects of clinical rehabilitation for splinting restorations between chronic periodontitis and a healthy periodontium. In addition to being free of periodontal disease, none of these patients were missing teeth in the nine units of the anterior segment of one jaw. The patients abided by the same exclusion criteria as the periodontally-involved patients.

### Splinting materials

One operator directly fabricated all restorations using a quartz fiber splint [Quartz Splint Woven(0.3 × 2.5 mm), Recherches Techniques Dentaires (RTD), Saint Egreve, France] in combination with flowable resin (Quartz splint resin, RTD, Saint Egreve, France; Filtek^™^ Z350 XT Flowable Restorative, 3M ESPE). Fiber posts (RelyX^™^ Fiber Post, 3M ESPE) were applied to each tooth that received a root canal treatment with FRC bonding. Resin (Filtek^™^ Z350 XT Universal Restorative, 3M ESPE) veneers were applied for the SV treatment.

### Operating procedure

#### Groups of restorations

**Figure 1:**
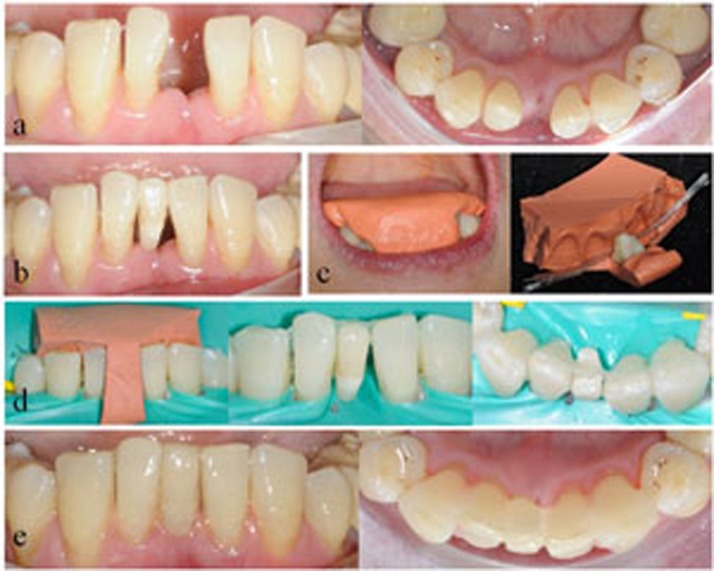
Direct view of clinical rehabilitation for a patient in the SB2 group. a) Frontal and lingual view of the lower dentition with missing left central incisor tooth; b) the extracted tooth prepared for the pontic was temporarily fixed in the suitable position by a thin layer of flowable composite resin without adhesive; c) the putty (3M, ESPE) silicone rubber template was prepared, and the pontic and quartz splint was laid; d) depending on placement of the silicone rubber template in the dentition, the pontic and quartz splint adhesive was applied at the corresponding position; e) frontal and lingual view of the final restoration with a direct resin veneer on the pontic

**Figure 2:**
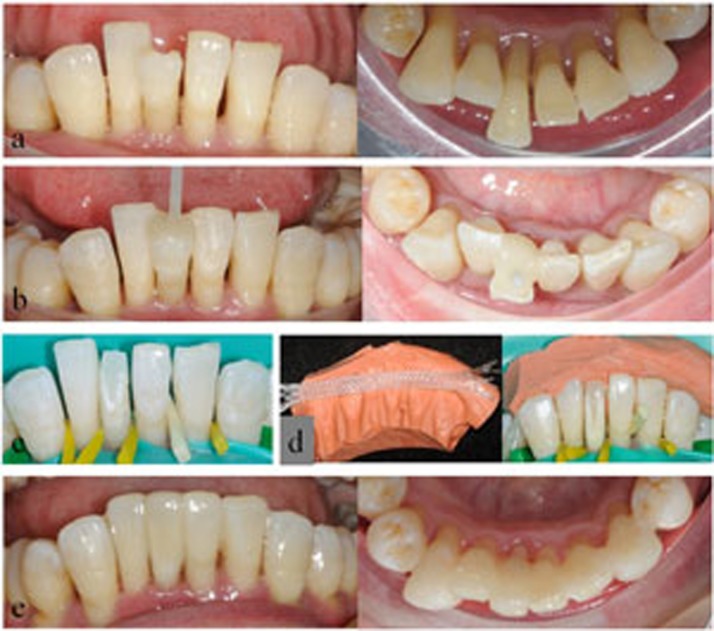
Direct view of clinical rehabilitation for a patient in the SV1 group. a) Frontal and lingual view of the lower dentition with severe facial dislodgement of the right central incisor tooth; b) tooth restored with fiber post and dual-cure resin core material; c) the dislodged tooth was modified to restore a normal arch form; d) The putty (3M, ESPE) silicone rubber template was prepared and placed in the dentition with a quartz splint adhesive at the corresponding position; e) frontal and lingual view of the final restoration with a direct resin veneer on the dislodged tooth

**Figure 3:**
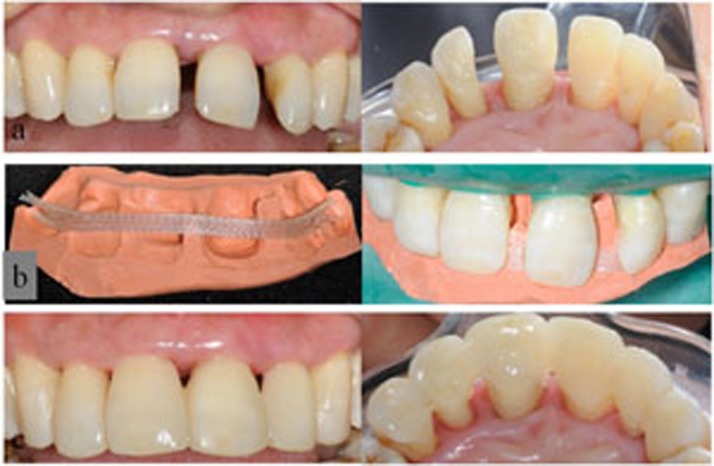
Direct view of the clinical rehabilitation for a patient in the SV2 group. a) Frontal and palatal view of the upper dentition with disorder space between the central incisor teeth and left lateral incisor tooth; b) the putty (3M, ESPE) silicone rubber template was placed in the dentition with a quartz splint adhesive in the corresponding position; c) frontal and lingual view of the final restoration with direct resin veneers on the incisor teeth and left lateral incisor tooth

**Figure 4:**
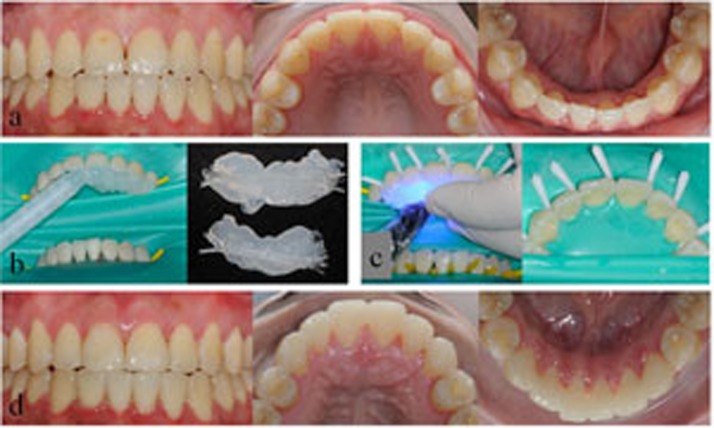
Direct view of the clinical rehabilitation for a patient in the OS group. a) Frontal and occlusal view of both upper and lower dentitions; b) the transparent silicone rubber (Bisco Inc., USA) templates were prepared, and the quartz splints were laid; c) the transparent silicone rubber template was placed and photo-polymerised in the upper dentition with a quartz splint adhesive in the corresponding position and fit well; d) frontal and lingual view of the final restorations

Each unit of restoration was applied from canine to canine in the anterior segment of one jaw. As shown in Table 1, four types of splinting restorations were applied and studied: restorations of FRC-bonded splint-bridges (SB; SB1, SB in combination with fiber posts; SB2, SB without fiber posts); combined restorations of FRC-bonded splint and resin veneer (SV; SV1, combined restorations of FRC-bonded splint, resin veneer and fiber posts; SV2, combined restorations of FRC-bonded splint and resin veneer without fiber posts); restorations of FRC-bonded splint (S; S1, S in combination with fiber posts; S2, S without fiber posts); and orthodontic retainers with FRC-bonded splints (OS) as a control for S2 to evaluate the effects of clinical rehabilitation. The operating procedure and direct-viewing effects of clinical rehabilitation for the SB2, SV1, SV2 and OS groups are shown in Figures 1,2,3,4.

#### Tooth preparation

The teeth for splinting were cleaned with a pumice using a prophylactic brush. When the restoration was planned on the maxilla, the lingual surfaces needed to be ground to allow for a 0.5 mm interspace. Teeth that had undergone root canals were treated with fiber-post restorations.

For SV, the configurations of the coronals of the teeth with severe facial-lingual dislodgement were modified to restore a normal arch form with fiber posts and dual-cure resin core materials (Paro Core, coltène/whaledent, Swiss). For SB, extracted teeth with rimmed roots and pulp spaces were obturated with resin core or commercial acrylic resin (Heraeus, German) with grooves (0.5 × 3 mm) on the lingual surface to inlay the splint and process the area for a pontic. The pontic was prepared for a modified ridge lap-type with a slight concavity that conformed to a residual ridge^32^ and was temporarily fixed in the suitable position by a thin layer of flowable composite resin (Filtek^™^ Z350 XT Flowable Restorative, 3M ESPE) which was photo-polymerided for 20 seconds without adhesive. A silicone rubber template was then prepared by placing silicone rubber on the lingual and incisal surfaces of all bonded teeth and covering the labial surface of the pontic. For the SV, S and OS treatments, the silicone rubber template was placed on the lingual and incisal surfaces of all bonded teeth.

#### Bonding procedure

All splints were applied from canine to canine and prepared under a rubber dam. The enamel surfaces on the palatal or lingual side were subsequently etched with 35% orthophosphoric acid (Gluma Etch, Heraeus, Genman) for 60 seconds. After rinsing with water and air drying, the corresponding intermediate adhesive (One-Step Plus filled universal dental adhesive, Bisco Inc., USA) was applied to the surfaces using a microbrush and photo-polymerised for 10 seconds. With the aid of corresponding silicone rubber template, a previously measured length of the single-layer quartz splint and the flowable resin (Quartz splint resin, RTD) were then placed on the lingual surface of the tooth to continuously align the FRC. For SB, the extracted tooth or commercial acrylic resin tooth for the pontic was placed in the silicone rubber template to be adhered to quartz splint and neighbouring teeth in the correct position. After a preliminarily photo-polymeridation for 20 seconds, the silicone rubber template was removed to allow the entire FRC to fully polymerise from the lingual aspect. The exposed surfaces of the FRC were then covered with another thick flowable resin composite (Filtek^™^ Z350 XT Flowable Restorative, 3M ESPE) and photo-polymerised again for 40 seconds from all aspects. Direct resin veneers were applied for SV when the facial view needed to be improved. Finally, after the removal of the excess resin composite and occlusal adjustments, the composite surfaces were polished with coarse, medium, fine, and ultrafine finishing disks (Sof-Lex^™^, 3M ESPE) in sequence in order to not expose the fibers. The restorations were cleaned and polished at each recall and repaired in case of fracture or the debonding of the splints or resin.

### Clinical evaluation

The patient satisfaction was assessed only at baseline. However, the clinical rehabilitation and periodontal condition were first assessed at baseline (immediately after splinting procedures) and thereafter annually during the follow-up visits according to the written protocol. The patients were informed about possible complications and instructed to call upon experience of a failure that could occur before the actual annual follow-up appointment.

#### Patient satisfaction survey

Sixty-nine patients were interviewed regarding their satisfaction with their restorations (regarding general satisfaction) using a visual analogue scale (VAS) of 100 mm with the endpoints extremely dissatisfied (0) and extremely satisfied (100).^33^

#### Effects of clinical rehabilitation

The modified USPHS criteria^34^ (Table 2) were used to evaluate retention, marginal integrity, marginal discolouration, anatomic form and secondary caries in all groups (SB, SV, S and OS) at baseline, 1, 2, 3 and 4 years after splinting. The mobility of the natural teeth in the SB, SV and S groups was evaluated according to Miller's classification^31^ at baseline, 1, 2, 3 and 4 years after splinting.

#### Periodontal evaluation

The periodontal pocket depth (PPD) and clinical attachment level (CAL) were measured at six sites (mesiobuccal, buccal, distobuccal, distolingual, lingual and mesiolingual sites) on each natural tooth of each restoration unit in the SB, SV and S groups at baseline, 1, 2, 3 and 4 years after splinting. The SB group included 30 sites per restoration, while the SV and S groups included 36 sites per unit. Williams periodontal probes with 1, 2, 3, 5, 7, 8, 9 and 10 mm markings^35^ were used.

### Statistical analysis

A statistical analysis was performed using the SPSS statistical package 13.0 (IBM Corporation, Armonk, NY, USA). A restoration unit comprised each study unit. The effects of clinical rehabilitation were analysed with a chi-square test. The PPD and CAL data are expressed as means ± standard deviation (SD). The PPD and CAL were analysed with a factorial-designed analysis of variance (ANOVA). The differences were considered to be significant when the p-value did not exceed 0.05.

## Results

### General satisfaction

VAS evaluation of general satisfaction at baseline showed that 59 (85.5%) patients scored the restorations 90 and 100, and 10 (14.5%) patients scored them 80 and 90 (mean VAS score of 92.4 ± 4.2).

### Effects of clinical rehabilitation

In total, none of the patients dropped out, and four recalls were performed for all patients. For the 63 periodontal patients, none of the anterior teeth in the opposing dentition of each restoration unit were mobile after the initial periodontal therapy, and none of the teeth had undergone additional splinting restorations. All patients received physiological occlusion after splinting restoration. All restorations resulted in good aesthetic outcomes. The evaluations of clinical rehabilitation are summarised in Table 3, and no caries were found within or adjacent to any of the splints. All restorations had a 100% acceptable rating scale for each category at the first recall, and SV1, SV2, S1 and OS all scored 100% on an acceptable rating scale (Alpha and Bravo) for each category that was directly evaluated at each recall. Both SB1 and S2 were rated 100% acceptable on retention and anatomic form at each recall. However, both the marginal integrity and the marginal discolouration scores were 88.9%, 88.9%, and 88.9% for SB1 and those were 84.6%, 76.9% and 76.9% for S2 when measured 2, 3 and 4 years after splinting, respectively. For SB2, when measured 2, 3 and 4 years after splinting, both the retention and anatomic form scores were 84.6%, 86.4% and 76.9%, while both the marginal integrity and the marginal discolouration scores were 76.9%, 69.2% and 69.2%. All restorations for the SB1, SB2 and S2 treatment groups that received an unacceptable rating (Charlie) were conducted in the maxilla. As these consisted of different restoration types which were not equivalent in our study, we did not think it was appropriate to use any analytical statistics to compare parameters between groups. However, as Table 3 showed, the value of acceptability ratings for retention, anatomical form, marginal integrity and marginal discolouration were higher for SB1 than for SB2 at 2, 3 and 4 years after splinting. Similarly, the value of marginal integrity and marginal discolouration scores were higher for S1 than S2 at the same time points.

The mobilities (M) of all natural teeth in the SB, SV and S groups at baseline and the mobility of all natural teeth in the SB1, SV and S at each recall were M0. In two restoration units of the SB2 group at the three-year recall and 3 restoration units of SB2 group at the four-year recall, the natural teeth returned to their pre-splinting mobility due to fracture and the debonding of splints, which resulted in an unacceptable rating (Charlie) for the retention of corresponding splinting restorations.

### Periodontal evaluation

**Figure 5:**
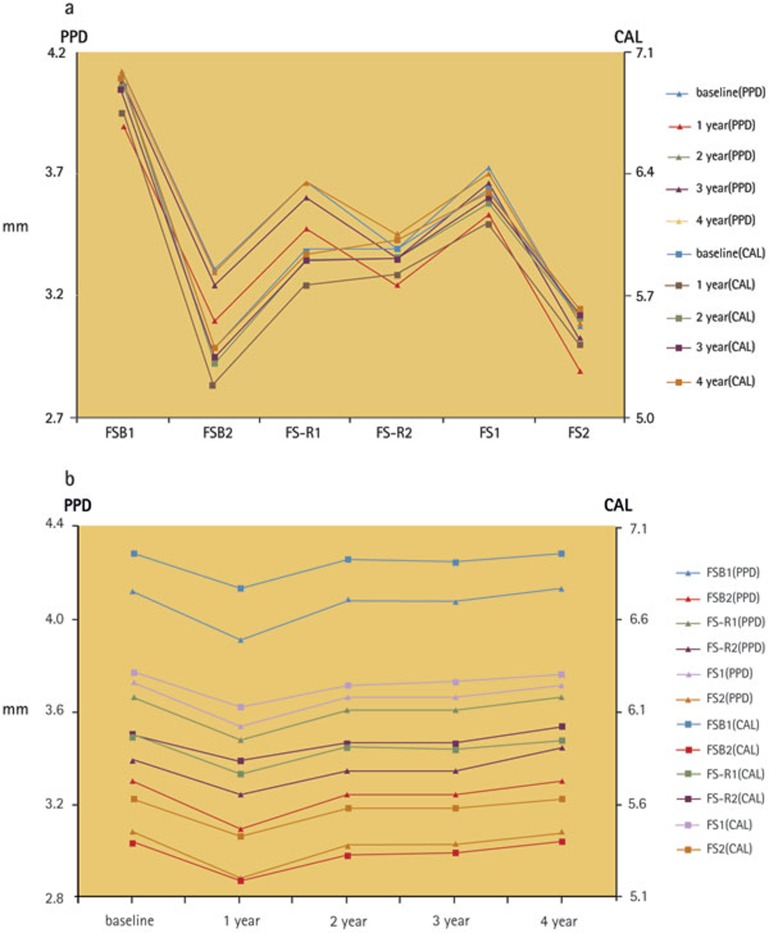
a) The mean PPD and CAL measured in different groups (9 patients in SB1, 13 patients in SB2, 9 patients in SV1, 9 patients in SV2, 10 patients in S1 and 13 patients in S2) at baseline as well as 1, 2, 3 and 4 years later; b) the changes at baseline as well as 1, 2, 3 and 4 years later within the same group

The total mean PPD for the splinted natural teeth was 3.4 ± 1.0 mm, and the total mean CAL was 5.9 ± 1.2 mm. The mean PPD and CAL of the different groups are shown in Figure 5a, and their changes over time within the same group are shown in Figure 5b. The mean PPD was 3.5 ± 1.0 mm at baseline and then decreased to 3.3 ± 1.0 mm at the one-year recall (p <0.05). It then increased to 3.5 ± 1.0 mm again at the four-year recall (p >0.05). This change was similar to that of the mean CAL. Neither the PPD nor the CAL differed between baseline and the two-year, three-year recall or four-year recall (p >0.05).

## Discussion

Aesthetic restoration for periodontally-involved anterior teeth is a challenge. In this retrospective study, we applied a combination of an FRC-bonded splint, bridge, fiber post and/or resin veneer in prosthodontic treatment for periodontally-involved tooth mobility, chronic anterior tooth dislodgement or anterior tooth loss. Under the limits of this retrospective evaluation, this approach resulted in acceptable outcomes for all groups and minimally invasive approaches for the restoration in the aesthetic zone of periodontal patients.

In our study, the periodontally-involved anterior teeth presenting with hypermobility (MII or above) after initial periodontal therapy received root canal treatments to prevent combined periodontic-endodontic lesions after restoration and sensitivity caused by gingival recession. The periodontic lesions of these teeth were usually in the apical third of the root, and most lesions were close to the root apical region. This approach constituted an empirical clinical therapy that blocked the possibilities of periodontic-endodontic infection. Traditionally, periodontally involved teeth do not usually need root canal treatments except when associated with endodontic lesions because keeping the pulp as vital as possible was a basic principle for dental treatments. Many studies showed endodontically treated teeth without mobility were generally weaker due to loss of tooth structure and removal of the pulp, and consequentially fractures often occurred in pulpless teeth.^36^,^37^,^38^ However, we had not reviewed the related literature comparing the tooth longevity of periodontally-involved natural teeth with and without root canal treatments. In our opinion, tooth fracture would not have occurred among periodontally involved teeth presenting with hypermobility, so the tooth longevity would not be affected by whether or not root canal treatments were received. Based on this opinion, another aim of root canal treatments in this study was to provide convenience for the aesthetic restoration. The teeth with severe facial-lingual dislodgement received root canal treatments because they required significant tooth reduction. Posts are often required to restore endodontically treated teeth in order to retain and provide resistance for the core material, to provide corono-radicular stabilisation and to facilitate the transmission of the dental loads from the coronal structure to the root.^39^ In our study, glass fiber posts were applied because of their advantageous features, including their aesthetic effects and elastic behaviour similar to that of dentine, which permitted a uniform stress distribution within the root.^40^,^41^ The fiber posts connected the tooth dentin and the FRC-bonded splint and/or resin veneers to retain and provide resistance for the splinting restorations.

The splinting method was convenient and distinctive when employed with the aid of a self-made silicone rubber template of putty or heavy body, which has been rarely reported. The silicone rubber for the template, which might be transparent or non-transparent, was placed on the lingual and incisal surfaces of all bonded teeth and covered the labial surface of the pontic for impression in the SB group, while it was only placed on the lingual and incisal surfaces of all bonded teeth in the SV, S and OS groups. The splint was preliminarily photo-polymerised from the labial and proximal aspects of interdental spaces if the silicone rubber template was non-transparent. The silicone rubber template not only laminated the bonded lingual surfaces of the splinting teeth with the splint, but also ensured that the pontic adhered to the splint and to neighbouring teeth in the correct position. These advantages simplified the FRC-bonded splint-bridges or splint procedures.

Although the patient satisfaction with all combined splinting restorations was generally high, which could be partly because of this being single visit treatment, the clinical rehabilitation outcomes measured in our study were all satisfactory. All restorations resulted in good aesthetic outcomes. SV1, SV2, S1 and OS all showed 100% acceptable ratings in each category that was directly evaluated at each recall; this indicated that periodontally-involved anterior teeth were successfully rehabilitated for up to four years using an FRC-bonded splint and resin veneer combination or an FRC-bonded splint and fiber post combination. The FRC-bonded splints also successfully performed as orthodontic retainers in the healthy periodontal negative controls. This success was closely related to the good flexure and rigidity of the quartz fiber splints. In our study, all restorations were made of single-layer woven quartz splints, whose flexural strength exceeds 120 MPa. Moreover, a study of the rigidity of quartz-fiber splints by Berthold *et* al. showed that reinforcement materials significantly influenced splint rigidity. Woven quartz splints are rigid quartz fiber splints that can be used to treat horizontal root fractures and alveolar process fractures.^42^ However, the rigidity of single-layer woven quarts splints could not satisfy all of our needs for splint-based restoration, especially for FRC-bonded splint-bridges. In our study, SB2 received the lowest value of acceptability ratings. Thus, we will consider the use of two or more layers of woven quartz splints in future studies to increase the rigidity if needed. Here, the thickness of a single-layer never exceeded 0.3 mm, while quartz splints could be placed on both the labial and lingual sides in combination with resin veneers for smaller interspaces.

In this study, FRC-bonded splints without fiber posts for periodontally involved teeth received 100% acceptable ratings for retention and anatomic form but 76.9% ratings for marginal integrity and marginal discolouration. When used as orthodontic retainers for healthy periodontal controls, FRC-bonded splints received 100% acceptable ratings for each category. This difference indicated that tooth mobility significantly negatively impacts the clinical success rates of splinting restorations. Our evaluation of clinical rehabilitation showed that these unacceptable ratings all appeared in the maxilla, which indicated that the FRC materials on the lingual surfaces of periodontally-involved maxillary teeth were easily affected by occlusion, that is, easily wore off, debonded or fractured after improper chewing.

The acceptable rating value of splinting restorations with fiber posts exceeded that of the splinting restorations without fiber posts, which indicated that combining the restoration with fiber posts might help to improve the clinical success of the splinting restorations in the aesthetic zone by providing retention and resistance for the splinting restorations. For the combination of an FRC-bonded splint with a resin veneer, the acceptable ratings were 100% for all outcomes, irrespective of the presence of a fiber post. We attributed this success to the increase in the area of resin adhesion by the resin veneer, which strengthened the entire prosthesis and may have improved clinical success rates of the splinting restorations.

In a pilot study, Kumbuloglupilot *et al*. evaluated the performance of FRC splints for the treatment of periodontally-involved mandibular anterior teeth and concluded that direct tooth splinting with E-glass FRC material performed successfully for up to 4.5 years; the periodontal status of the splinted teeth showed decreased PPD and CAL values.^25^ In contrast, the periodontal status of the splinted teeth in our study showed decreased PPD and CAL values only at the one-year recall. Others have reported that splinting could increase patient comfort during chewing when used to connect periodontally compromised teeth; however, these splints also hinder oral hygiene procedures.^43^ PPD and CAL are important indicators of oral hygiene. In our study, decreased PPD and CAL values were observed only at the one-year recall; these values did not differ between the baseline and the two, three- or four-year recall. We believe that these parameters were controlled by oral hygiene. In general, the patients paid great attention to maintaining their oral hygiene and protecting the restorations within the first year of splinting, which was supported by the results of our clinical and periodontal evaluations. Over time, the patients required reminders to maintain enhanced oral hygiene measures, regularly perform periodontal maintenance, and prevent improper chewing. Quartz FRC-bonded splint-bridges or splints did not show an increased risk of PPD and CAL when the periondontally involved teeth were annually evaluated at follow-up.

In general, combinations of an FRC-bonded splint, bridge, fiber post and/or resin veneers for prosthodontic treatment in the aesthetic zone of periodontal patients resulted in good clinical rehabilitation as well as good periodontal status without an increased risk of PPD and CAL. However, the key to maintaining these clinical effects up to four years following treatment was high patient attention to oral hygiene and the protection of the restorations. Combining a fiber post and resin veneer may help improve the clinical success rates of splinting restorations in the aesthetic zone when using self-made silicon rubber templates, which simplify splinting procedures. In future studies, we will consider the use of two or more layers of woven quartz splints to increase the rigidity of splinted restorations if needed. For smaller interspaces, the quartz splints may also be placed on both the labial and lingual sides and combined with resin veneers. In addition to the treatment of periodontal patients, a combination of an FRC-bonded splint, bridge, fiber post and/or resin veneer for prosthodontic treatment may also be used as a prosthetic after orthodontic correction, to treat anterior teeth trauma, etc.

In conclusion, combining an FRC-bonded splint, bridge, fiber post and/or resin veneer for minimally invasive prosthodontic treatment in the aesthetic zone is a good choice for periodontal patients.

## Financial support

This work was supported by grants from the Natural Science Foundation of China for young scholars (No 81300908), the Fundamental Research Funds for the Central Universities (No 21615480), Guangdong Provincial Technology and Research Project (No 2012B061700091) and Science Research Project of Traditional Chinese Medicine Bureau of Guangdong Province (No 20141068).

## Figures and Tables

**Table 1:** General condition of restoration units

	SB		SV		S		OS
	SB1	SB2	SV1	SV2	S1	S2	
Upper	3	3	6	4	3	4	6
Lower	6	10	3	5	7	9	3
Total	9	13	9	9	10	13	9
Each unit of restoration was applied from canine to canine at the anterior segment of one jaw.							
Abbreviations: SB, FRC bonded splint-bridges; SV, combined restorations of FRC bonded splint and resin veneer; S, FRC bonded splints; SB1, SB in combination with fiber posts; SB2, SB without fiber posts; SV1, combined restorations of FRC bonded splint, resin veneer and fiber posts; SV2, combined restorations of FRC bonded splint and resin veneer without fiber posts; S1, S in combination with fiber posts; S2, S without fiber posts; OS, orthodontic retainer with FRC bonded splints.							

**Table 2:** Modified USPHS criteria rating system

Category	Rating scale		Criteria
	Acceptable	Unacceptable	
Retention	Alpha (A)	Charlie (C)	Restoration is presentRestorations is partially or totally lost
Marginal integrity	Alpha (A)Bravo (B)	Charlie (C)Delta (D)	No visible gap in which the explorer will penetrateThere is visible gap, the explorer will penetrate or catchThe explorer penetrates the gap and dentin or base is exposedThe restoration is mobile, partially or totally fractured or lost
Marginal discolouration	Alpha (A)Bravo (B)	Charlie (C)	No discolourationDiscolouration is present but has not penetrated along the marginDiscolouration has penetrated along the margin
Anatomic form	Alpha (A)Bravo (B)	Charlie (C)	Restoration is continuous with existing anatomic formRestoration is discontinuous with existing anatomic form, but dentin or base is not exposedSufficient material is lost to expose dentin or base
Secondary caries	Alpha (A)	Charlie (C)	No caries is present at the margin of the restorationThere is evidence of caries at the margin of the restoration

**Table 3:** Summary of direct evaluations of clinical rehabilitation

Category	Group	Baseline	1 year	2 years	3 years	4 years
		%A + B	%A + B	%A + B	%A + B	%A + B
Retention	SB1	100%(9/9)	100%(9/9)	100%(9/9)	100%(9/9)	100%(9/9)
	SB2	100%(13/13)	100%(13/13)	84.6%(11/13)	84.6%(11/13)	76.9%(10/13)
	SV1	100%(9/9)	100%(9/9)	100%(9/9)	100%(9/9)	100%(9/9)
	SV2	100%(9/9)	100%(9/9)	100%(9/9)	100%(9/9)	100%(9/9)
	S1	100%(10/10)	100%(10/10)	100%(10/10)	100%(10/10)	100%(10/10)
	S2	100%(13/13)	100%(13/13)	100%(13/13)	100%(13/13)	100%(13/13)
	OS	100%(9/9)	100%(9/9)	100%(9/9)	100%(9/9)	100%(9/9)
Marginal integrity	SB1	100%(9/9)	100%(9/9)	88.9%(8/9)	88.9%(8/9)	88.9%(8/9)
	SB2	100%(13/13)	100%(13/13)	76.9%(10/13)	69.2%(9/13)	69.2%(9/13)
	SV1	100%(9/9)	100%(9/9)	100%(9/9)	100%(9/9)	100%(9/9)
	SV2	100%(9/9)	100%(9/9)	100%(9/9)	100%(9/9)	100%(9/9)
	S1	100%(10/10)	100%(10/10)	100%(10/10)	100%(10/10)	100%(9/10)
	S2	100%(13/13)	100%(13/13)	84.6%(11/13)	76.9%(10/13)	76.9%(10/13)
	OS	100%(9/9)	100%(9/9)	100%(9/9)	100%(9/9)	100%(9/9)
Marginal discolouration	SB1	100%(9/9)	100%(9/9)	88.9%(8/9)	88.9%(8/9)	88.9%(8/9)
	SB2	100%(13/13)	100%(13/13)	76.9%(10/13)	69.2%(9/13)	69.2%(9/13)
	SV1SV2	100%(9/9)100%(9/9)	100%(9/9)100%(9/9)	100%(9/9)100%(9/9)	100%(9/9)100%(9/9)	100%(9/9)100%(9/9)
	S1	100%(10/10)	100%(10/10)	100%(10/10)	100%(10/10)	100%(9/10)
	S2	100%(13/13)	100%(13/13)	84.6%(11/13)	76.9%(10/13)	76.9%(10/13)
	OS	100%(9/9)	100%(9/9)	100%(9/9)	100%(9/9)	100%(9/9)
Anatomic form	SB1	100%(9/9)	100%(9/9)	100%(9/9)	100%(9/9)	100%(9/9)
	SB2	100%(13/13)	100%(13/13)	84.6%(11/13)	84.6%(11/13)	76.9%(10/13)
	SV1SV2	100%(9/9)100%(9/9)	100%(9/9)100%(9/9)	100%(9/9)100%(9/9)	100%(9/9)100%(9/9)	100%(9/9)100%(9/9)
	S1	100%(10/10)	100%(10/10)	100%(10/10)	100%(10/10)	100%(10/10)
	S2	100%(13/13)	100%(13/13)	100%(13/13)	100%(13/13)	100%(13/13)
	OS	100%(9/9)	100%(9/9)	100%(9/9)	100%(9/9)	100%(9/9)
Secondary caries	SB1	100%(9/9)	100%(9/9)	100%(9/9)	100%(9/9)	100%(9/9)
	SB2	100%(13/13)	100%(13/13)	100%(13/13)	100%(13/13)	100%(13/13)
	SV1SV2	100%(10/10)100%(9/9)	100%(10/10)100%(9/9)	100%(10/10)100%(9/9)	100%(10/10)100%(9/9)	100%(10/10)100%(9/9)
	S1	100%(9/9)	100%(9/9)	100%(9/9)	100%(9/9)	100%(9/9)
	S2	100%(13/13)	100%(13/13)	100%(13/13)	100%(13/13)	100%(13/13)
	OS	100%(9/9)	100%(9/9)	100%(9/9)	100%(9/9)	100%(9/9)
Abbreviations: A, Alpha; B, Bravo; SB1, FRC bonded splint-bridges in combination with fiber posts; SB2, FRC bonded splint-bridges without fiber posts; SV1, combined restorations of FRC bonded splint, resin veneer and fiber posts; SV2, combined restorations of FRC bonded splint and resin veneer without fiber posts; S1, FRC bonded splints in combination with fiber posts; S2, FRC bonded splints without fiber posts; OS, orthodontic retainer with FRC bonded splints						

## Notes

**Refereed Paper**
